# Dataset on the mechanical and physical characterization of the Ecuadorian *Guadua angustifolia kunth* bamboo culms belonging to “Caña Mansa” biotype

**DOI:** 10.1016/j.dib.2023.109969

**Published:** 2023-12-16

**Authors:** José Padilla, Wilson Guachamín-Acero, Víctor H. Guerrero, Willan Monar, Patricia I. Pontón, Marco V. Guamán

**Affiliations:** a Department of Mechanical Engineering, Escuela Politécnica Nacional, Quito 170109, Ecuador; b Department of Materials, Escuela Politécnica Nacional, Quito 170109, Ecuador

**Keywords:** Green materials, Natural resources, Structural materials, Ultimate strength, Density

## Abstract

This article presents a set of experimental data on the mechanical and physical properties of the Ecuadorian endemic bamboo species *Guadua angustifolia kunth* (*Guadua a.k.*), specifically the “Caña Mansa” biotype. The data on compressive, shear, tensile and bending strength, as well as the moisture content and density, were obtained by carrying out the corresponding tests following the ISO 22157:2019 standard. For this purpose, each bamboo culm examined was divided along its height into three sections that were thoroughly characterized. The equations used for the calculations of the mechanical and physical properties are described in detail for each test. Besides, the main mechanical properties of the characterized bamboo were compared to those of similar species reported in the literature. Property charts (compressive/tensile strength and modulus of rupture vs. density) were built to compare the Ecuadorian biotype evaluated with other classical and green materials by using appropriate software. These data give an insight into the valorization of natural structural materials harvested in the Americas for potential applications in different engineering fields, particularly for sustainable building.

## Graphical abstract

**Figure:**
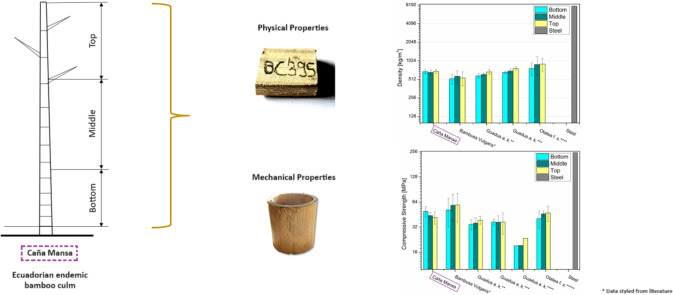

Specifications Table

**Table:** 

Subject	Materials characterization
Specific subject area	Natural structural materials
Data format	Raw, Analyzed
Type of data	Table, Figure
Data collection	Data on compression, shear and bending tests were acquired using a Tinius Olsen Super l-120 model universal testing machine, while data on tensile tests were obtained using a Shimadzu, UH-F500kNX model. According to the calibration certificates, the machines have at least 99.85 % accuracy for the range of forces considered in the tests. Physical data corresponding to density and moisture content were acquired employing a BIOBASE BOV-D30 electric oven (calibration range: 50–200 °C, accuracy ± 2 °C) and a digital balance (UX 1020H model, calibration range with external weights: 500–1020 g, accuracy 0.001 g), and a COBOS moisture meter MD-4 G (calibration range: 5–40 % moisture content), respectively. The figures were plotted using Origin 8.5.1 software. One-way analyses of variance (ANOVA) of the mechanical and physical properties were also performed employing Origin 8.5.1 software. CES EduPack 2019 software was used to build property charts to compare the mechanical strength of several materials. Literature was also used as data source.
Data source location	Location of bamboo culms (City/Town/Region): Jama/Manabí/Coastal region. Country: Ecuador. Latitude and longitude (GPS coordinates): 0° 07′ 31,0′’ S 80° 04′ 26,1′’ O 84 masl. The mechanical and physical characterizations were performed at Escuela Politécnica Nacional (EPN), Quito, Ecuador.
Data accessibility	Repository name: Mendeley Data Data identification number: 10.17632/dzccmchrtt.1 Direct URL to data: https://data.mendeley.com/datasets/dzccmchrtt/1

## 1 Value of the Data

• These data provide insights into the mechanical and physical properties of the “Caña Mansa” bamboo culms, which are especially relevant in construction and manufacturing fields. Thus, this information will allow researchers and engineers to design structural components through methods based on strengths by using this ecofriendly material.
• Although various bamboo species have been studied, including the tropical giant *Guadua a.k.*, the strongest bamboo family, there is a lack of data related to the mechanical and physical properties of a list of biotypes, particularly belonging to this last variety, such as “Caña Mansa”. Therefore, these data pave the way to elucidate how “Caña Mansa” endemic biotype stands out or aligns with other known varieties.
• The data on the characterization of endemic “Caña Mansa” bamboo, abundantly available in Ecuador, is of utmost importance to technify its use, preserve and promote the spreading of its crops, considering that this biotype presents the advantage of having no thorns on the basal and apical branches, being easier to handle in respect to other local biotypes. This supports the biodiversity conservation efforts in South American countries.
• Leveraging the mechanical and physical properties exhibited by the “Caña Mansa” biotype will be valuable for the local government to establish guidelines to optimize its consumption inside the country, and to promote both environmentally sustainable and inclusive development.
• The dataset on the mechanical properties of the endemic “Caña Mansa” bamboo biotype is key for the development of sheltered and cost-effective housing in earthquake-prone areas.

## 2 Background

The motivation for generating this dataset is to better understand the mechanical response to different loads of the Ecuadorian *Guadua a.k.* bamboo culms, belonging to “Caña Mansa” biotype, for improving their management and technical utilization, adding value to this native species. The methodology comprises the selection of the bamboo clumps, the collection and drying of the culms, the extraction and manufacturing of the specimens from three culm sections (bottom, middle, and top), following the ISO 22157:2019 standard. Finally, the data compilation was obtained after performing the corresponding tests to evaluate compressive, shear, tensile and bending strengths, as well as density and moisture content. This paper is also intended to demonstrate, through the data on mechanical and physical properties, that this ecofriendly material can be considered as an alternative candidate for structural sustainable construction. This goal is accomplished by comparing its mechanical properties and density, with those of other bamboo species and conventional construction materials commonly employed in this field.

## 3 Data Description

**Fig. 1:**
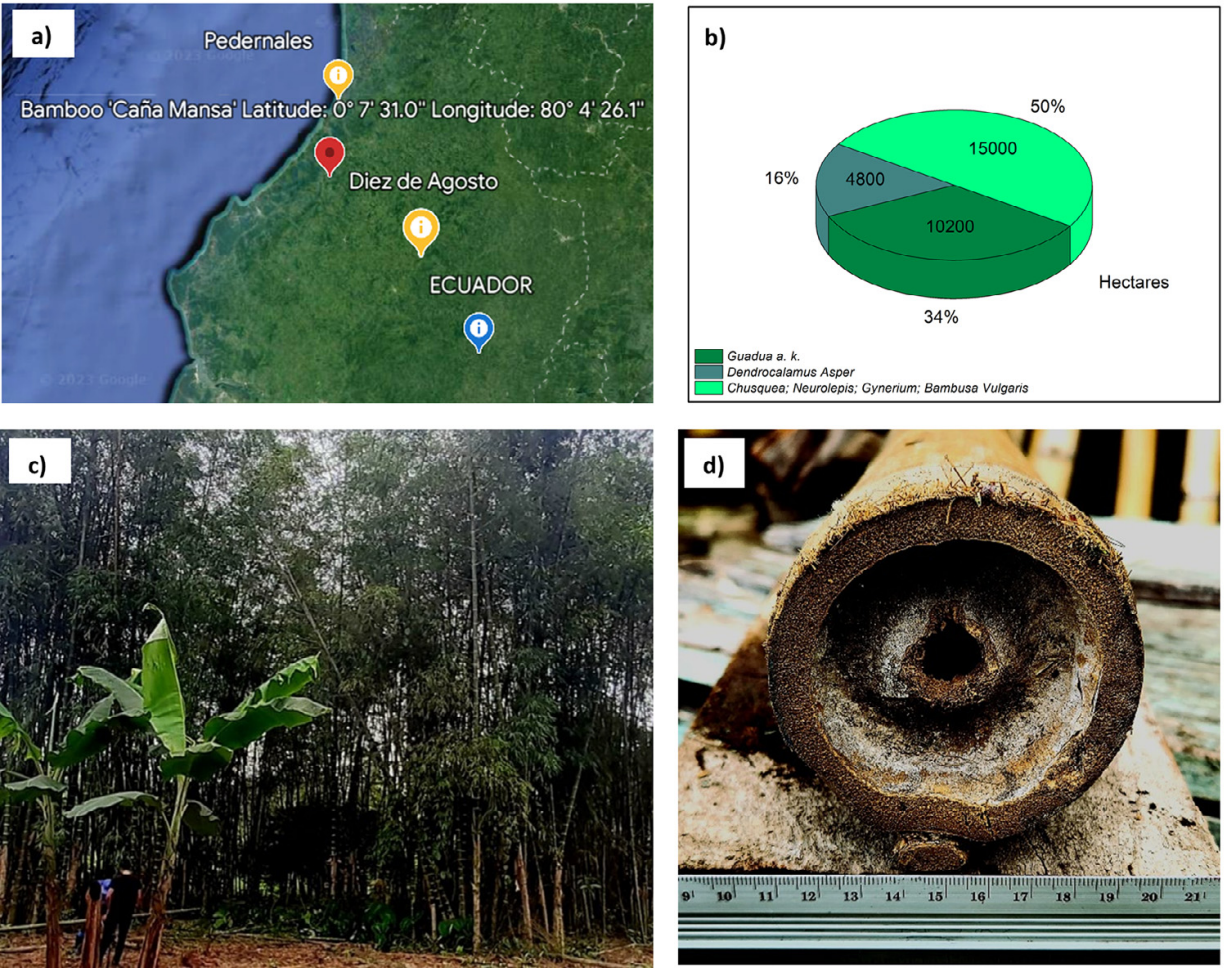
Collection of culms: a) geographical location, latitude and longitude shown using Google Earth, b) percentage per hectare of bamboo species cultivated in Ecuador styled from reference [5], note that “Caña Mansa” is a biotype of *Guadua a.k.*, c) one of the “Caña Mansa” bamboo clumps selected for the collection, and d) an average culm cross section (the mean diameter corresponds to 87.6 mm).

**Fig. 2:**
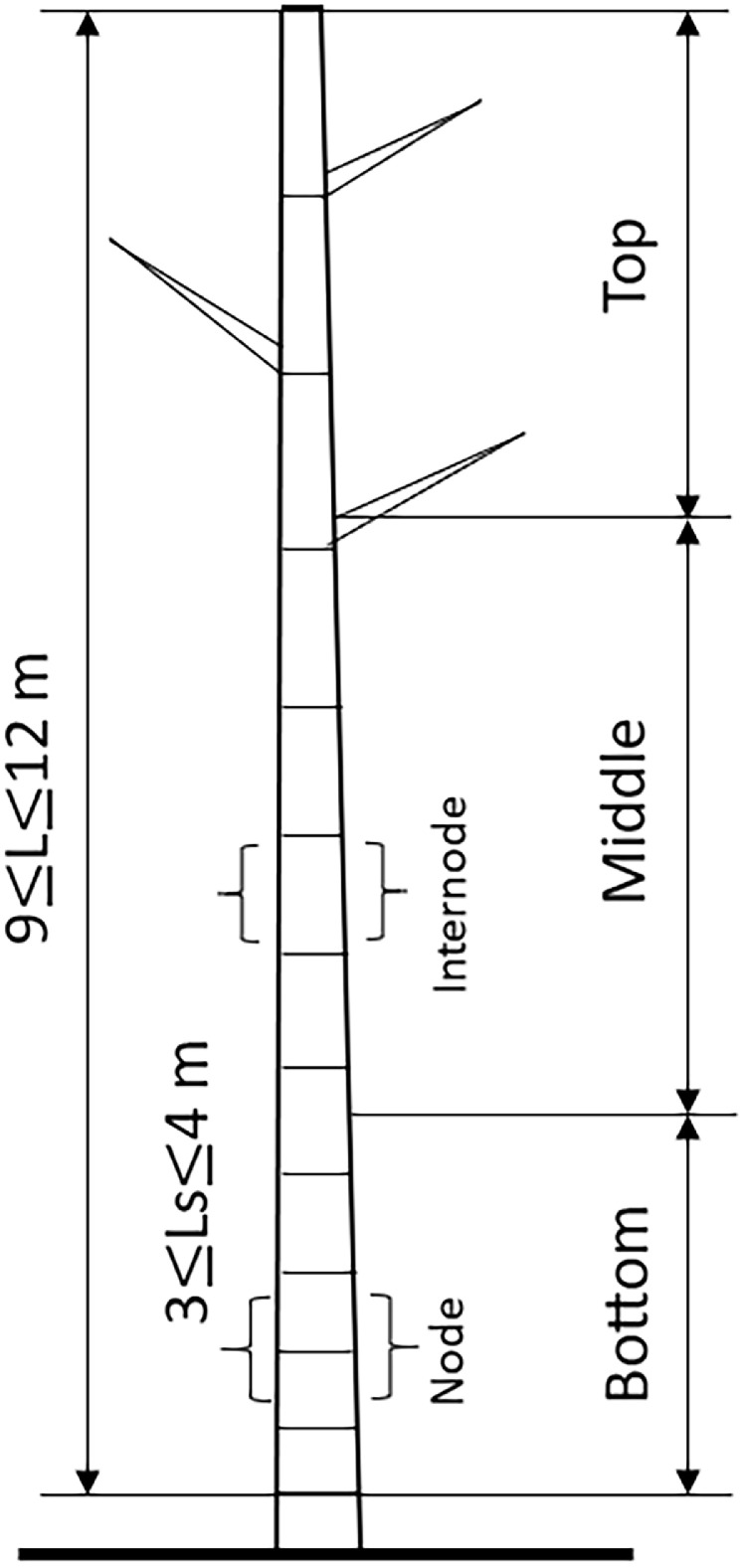
Total and section lengths of the “Caña Mansa” bamboo culms studied (“Ls” represents the length of the bottom, middle, and top sections, along the culm height).

**Fig. 3:**
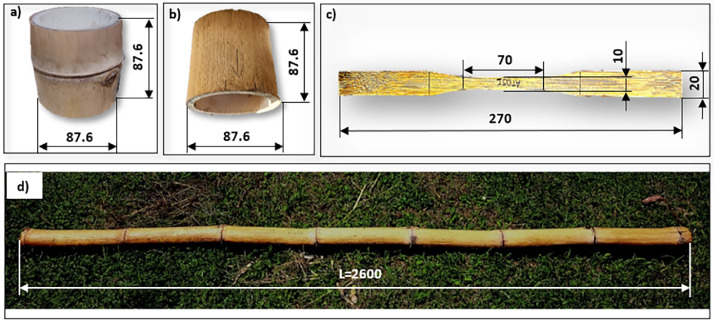
Images of the specimens used for a) shear test in the node section, b) shear test in the internode section (this type of specimen was also used for compression tests), c) tensile test in the node section (specimen for the tensile test in the internode section is not presented, but its geometry is the same), and d) bending test. The characterizations were performed according to the ISO 22157:2019 standard [1,2]. Dimensions are in mm.

**Fig. 4:**
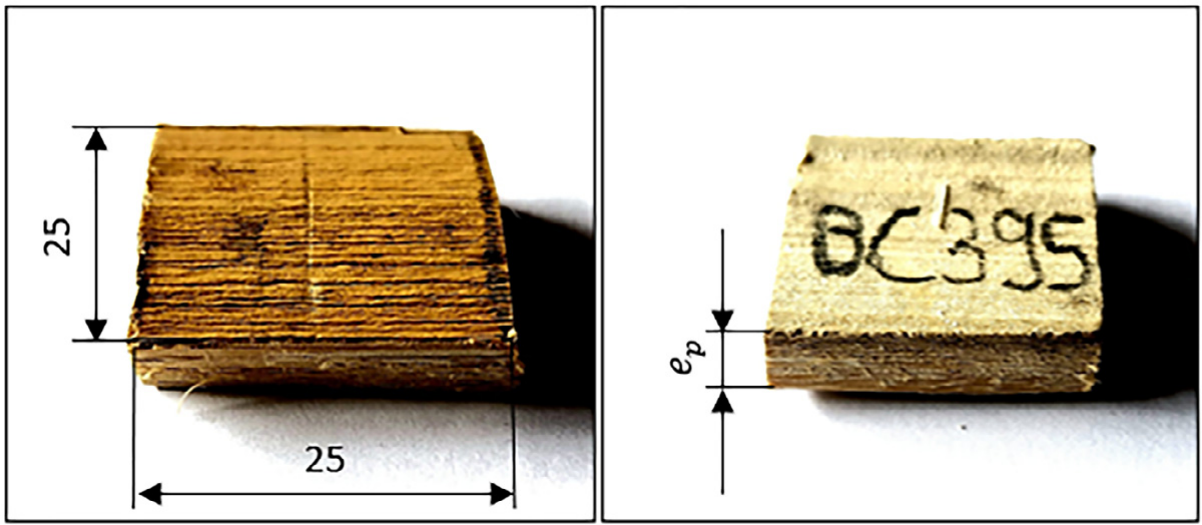
Images of the samples used for moisture content and density tests. Dimensions are in mm ($e_{p}$ is the thickness of the specimens used in the corresponding mechanical tests).

This paper presents, for the first time, the values of the main mechanical and physical properties of the “Caña Mansa” bamboo culms grown in Ecuador, belonging to the *Guadua a.k.* species. The dataset was obtained according to the ISO 22157:2019 standard [1,2]. Fig. 1 shows (a) the geographical location where the culms were collected, (b) the plantation distribution of different bamboo species in Ecuador per hectare, where the *Guadua a.k.* variety represents 34 % of the crops, (c) one of the bamboo clumps from which the culm samples were extracted, and (d) an average culm cross section after harvesting. A scheme of a bamboo culm, including the length of each culm section and the node and internode region (region without node), are depicted in Fig. 2. The images of the specimens used for mechanical testing, along with their geometry and dimensions, are detailed in Fig. 3. Note that the samples for the determination of the moisture content and density were obtained after performing the mechanical tests, from the part of the specimen nearest to the failure zone. These samples were manufactured with a rectangular prism shape ($25\times 25\times e_{p}$ mm), where $e_{p}$ is the wall thickness, as presented in Fig. 4. Table 1 summarizes the equations used for the calculation of the compressive, shear, tensile and bending strength (MOR), moisture content and density, according to ISO 22157:2019 standard [1,2]. The average ultimate strengths and their corresponding standard deviations, as well as the results of the one-way analysis of variance (ANOVA), are presented in Table 2. These data are given for the compressive, shear, tensile, and bending tests, and for each culm height section (bottom, middle, and top). One-way ANOVA was performed to gain a deeper understanding of the statistically significant changes in the mechanical properties of the bamboo specimens from the middle and top culms’ section, compared to the bottom one. The bottom section was arbitrarily selected as a reference for comparison of the mechanical properties. The probability value (p) was employed to evaluate the null hypothesis. If p≤0.05, there is a statistically significant change between the mean values of the property. Thus, the Tukey's multiple comparison test at 95 % confidence level was carried out. The corresponding increase or decrease on the mechanical property was indicated in parenthesis. Tables 3 and 4 show the computed average moisture content and density of each culm section, respectively. One-way ANOVA was also performed for the data on these physical properties to determine if a statistically significant difference arises for the middle or top sections compared to the bottom one.

**Table 1:** Equations used for the calculation of the mechanical and physical properties, according to the ISO 22157:2019 standard [1,2].

Test	Equation	N°	Variables and parameters
Compressive	$S_{uc}=\frac{F_{ult}}{A_{t}}$	(1)	$S_{uc}=$ compressive strength $A_{t}=$ cross-sectional area $F_{ult}=$ maximum load
Shear	$S_{us}=\frac{F_{ult}}{\sum (t\cdot l)}$	(2)	$S_{us}=$ shear strength $F_{ult}=$ maximum load t= wall thickness l= length
Tensile	$S_{ut}=\frac{F_{ult}}{A_{t}}$	(3)	$S_{ut}=$ tensile strength $F_{ult}=$ maximum load $A_{t}=$ mean cross-sectional area of the gauge
Bending	$S_{uf}=F_{ult}\cdot L\cdot \frac{D}{12I_{B}}$	(4)	$S_{uf}=$ modulus of rupture (MOR) $F_{ult}=$ maximum load L= free span or clear span D= outer diameter $I_{B}=$ second moment of area
Moisture content	$MC=\frac{m-m_{o}}{m_{o}}\cdot 100$	(5)	MC= moisture content m= mass of the test sample before drying $m_{o}=$ mass of the test sample after drying
Density	$\rho =\frac{m_{o}}{V}$	(6)	ρ= density $m_{o}=$ mass of the test sample after drying V= green volume of test sample

**Table 2:** Compressive, shear, tensile strengths, and MOR for the as-characterized “Caña Mansa” bamboo culms.

Test	Culm height section	Average [MPa]	ANOVA
Compressive	Bottom	49.55±7.01	
	Middle	44.02±4.41	▼ (11 %)
	Top	41.80±7.10	▼ (16 %)
Internode shear	Bottom	5.96±1.29	
	Middle	6.71±1.67	•
	Top	5.19±1.17	•
Node shear	Bottom	5.79±2.22	
	Middle	4.75±1.66	•
	Top	5.90±2.09	•
Internode tensile	Bottom	242.89±18.51	
	Middle	107.32±36.38	▼ (56 %)
	Top	187.98±47.21	▼ (23 %)
Node tensile	Bottom	110.38±22.71	
	Middle	66.43±29.78	▼ (40 %)
	Top	39.66±14.60	▼ (64 %)
Bending (MOR)	Bottom	60.15±6.13	
	Middle	69.16±12.26	•
	Top	88.23±33.04	▲ (47 %)

• The property of the culm height section is not significantly different from that of the bottom.

▼ The property of the culm height section is significantly lower than that of the bottom.

▲ The property of the culm height is significantly higher than that of the bottom.

**Table 3:** Moisture content of the “Caña Mansa” bamboo culm sections.

Test	Culm height section	Average [%]	ANOVA
Moisture content	Bottom	10.08±0.75	
	Middle	9.99±1.04	•
	Top	9.91±1.04	•

• Non-statistically significant difference between the moisture content of the culm height section and the bottom.

**Table 4:** Density of each “Caña Mansa” bamboo culm section.

Test	Culm height section	Average [kg/m^3^]	ANOVA
Density	Bottom	681.52±54.08	
	Middle	659.97±61.49	•
	Top	685.10±52.38	•

• Non-statistically significant difference between the density of the culm height section and the bottom.

**Fig. 5:**
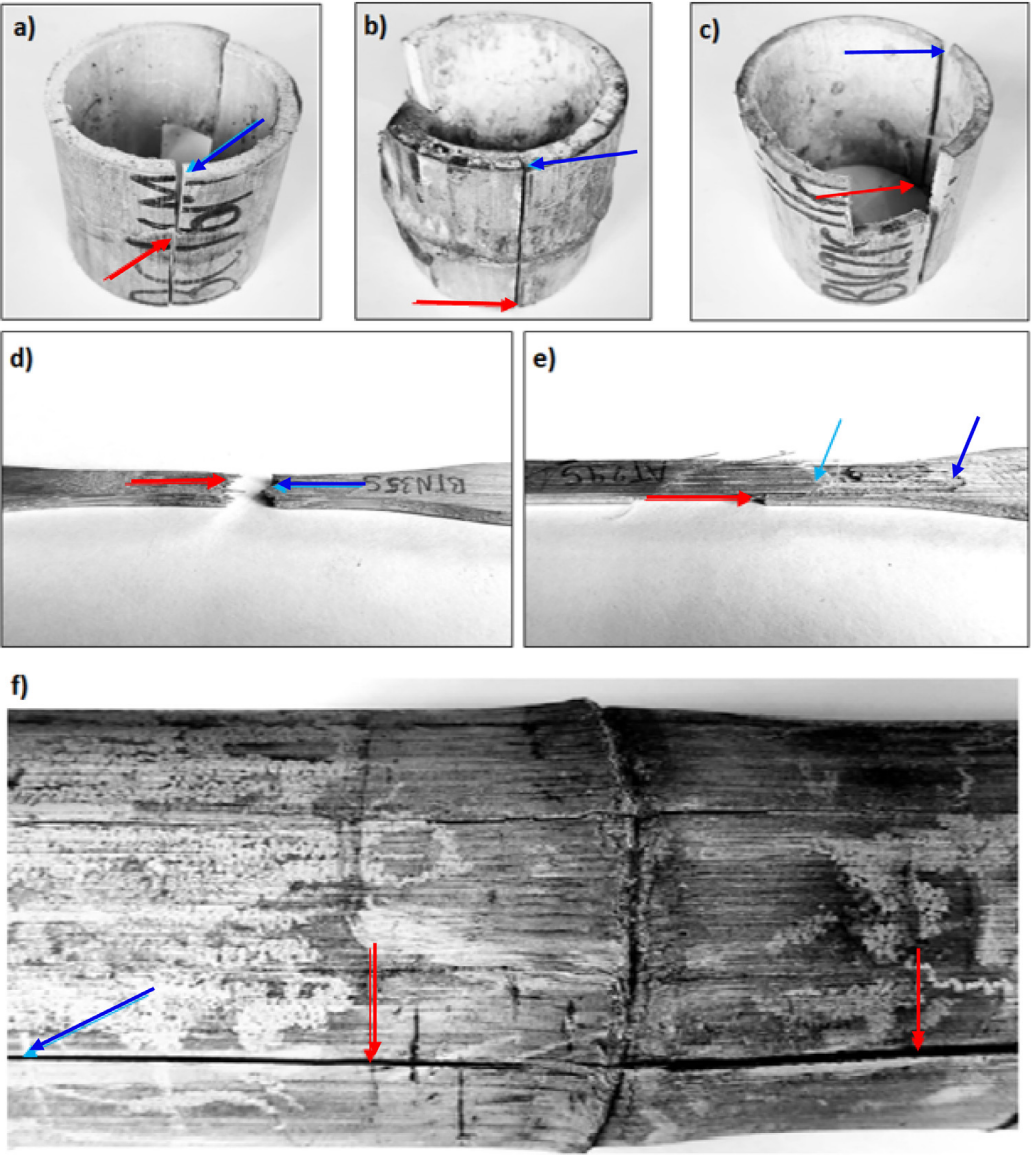
Failure modes exhibited by specimens after a) compression, b) node shear, c) internode shear, d) node tensile, e) internode tensile, and f) bending testing. The red arrows denote the initiation of the fracture crack, while the blue ones represent the end.

Fig. 5 depicts the failure zone that the as-characterized specimens exhibited at the end of the corresponding mechanical tests. For the compression specimens, the failure cracks typically initiated at the center of the specimen and propagated to the ends (Fig. 5a), as reported in the study conducted by Hamdan et al. [3]. The failure crack in the node and internode shear specimens initiated and propagated along maximum four cutting areas, determined by their length and thickness (Fig. 5b and c). In the tensile specimens the failure occurred at the node, while in the internode specimens, the beginning and the end of the crack are in different zones of the reduced section (Fig. 5d and e). For the bending specimens, the failure cracks initiated at the loading points and propagated longitudinally (Fig. 5f). These modes of failure were also described by Fabiani [4], where Italian bamboo culms belonging to *Phyllostachys Edulis* and *Phyllostachys Viridiglaucescens* species were characterized.

**Fig. 6:**
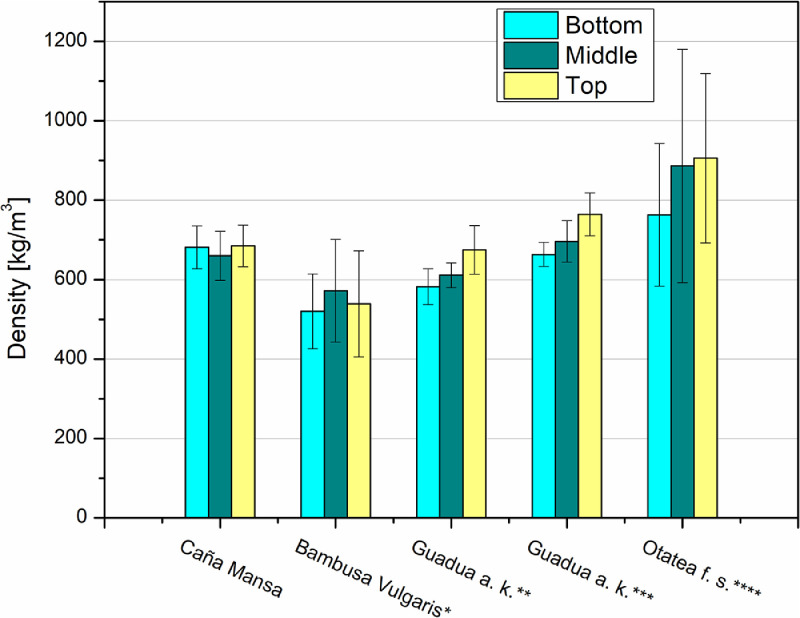
Comparison between the densities of the Ecuadorian “Caña Mansa” bamboo biotype and other bamboo species, harvested in Colombia and Mexico. Data styled from reference * [6], ** [7], *** [8], and **** [10].

**Fig. 7:**
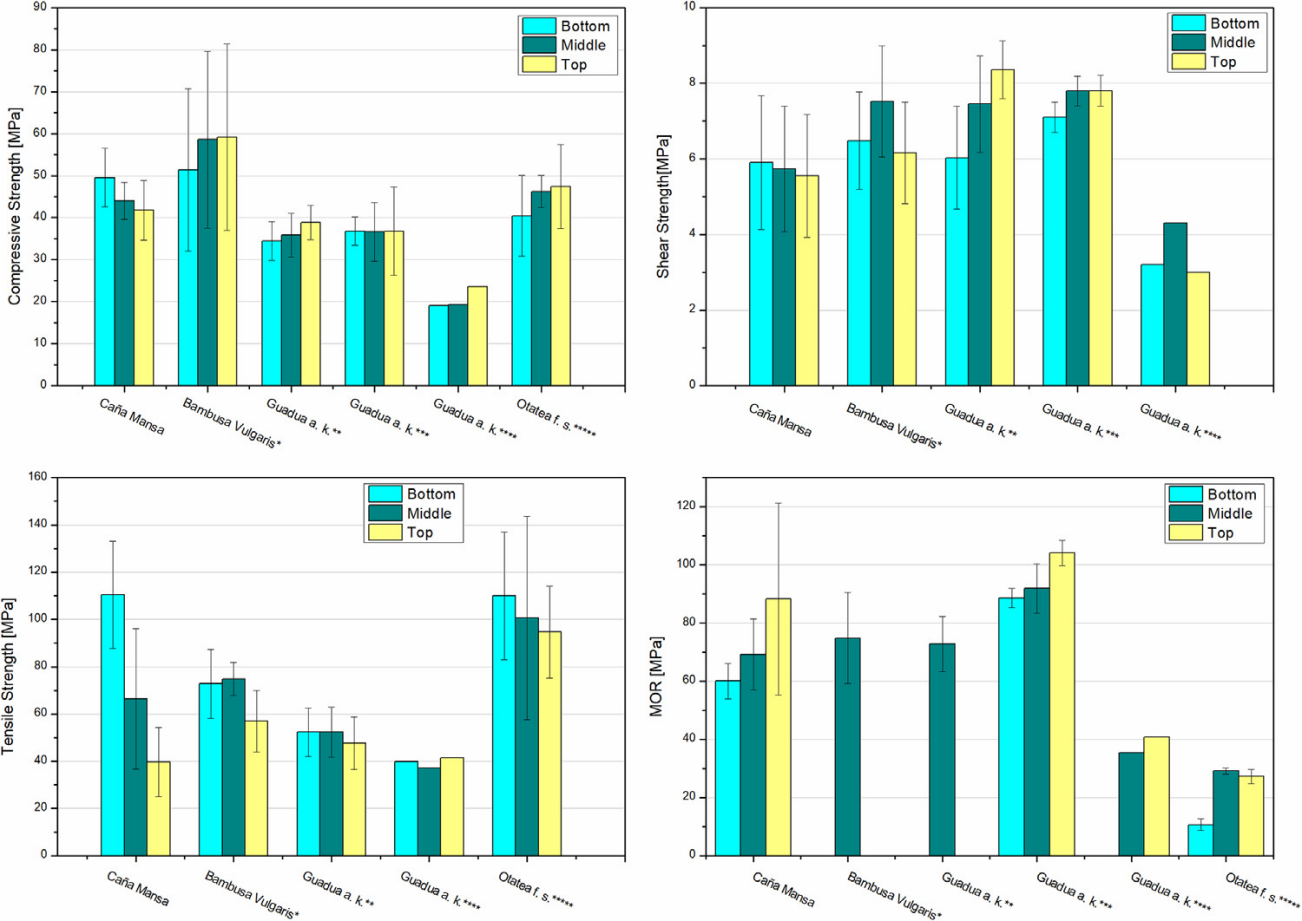
Comparison of the a) compression, b) tensile, c) shear strengths, and d) MOR of the Ecuadorian “Caña Mansa” bamboo biotype and other bamboo species, harvested in Colombia and Mexico. Data styled from reference * [6], ** [7], *** [8], **** [9], and ***** [10]. For tensile strength, only node specimens were included in the analysis to establish the comparison with benchmark studies.

The density and the ultimate strengths (in compression, shear, tensile, and bending) of the “Caña Mansa” bamboo culms were compared with other bamboo species, harvested in Colombia and Mexico, as presented in Figs. 6 and 7, respectively. Note that in Fig. 7, the average strength of the total specimens (including nodes and internodes) was contrasted with the literature. However, only node specimens were compared for the tensile test based on the reference studies.

**Fig. 8:**
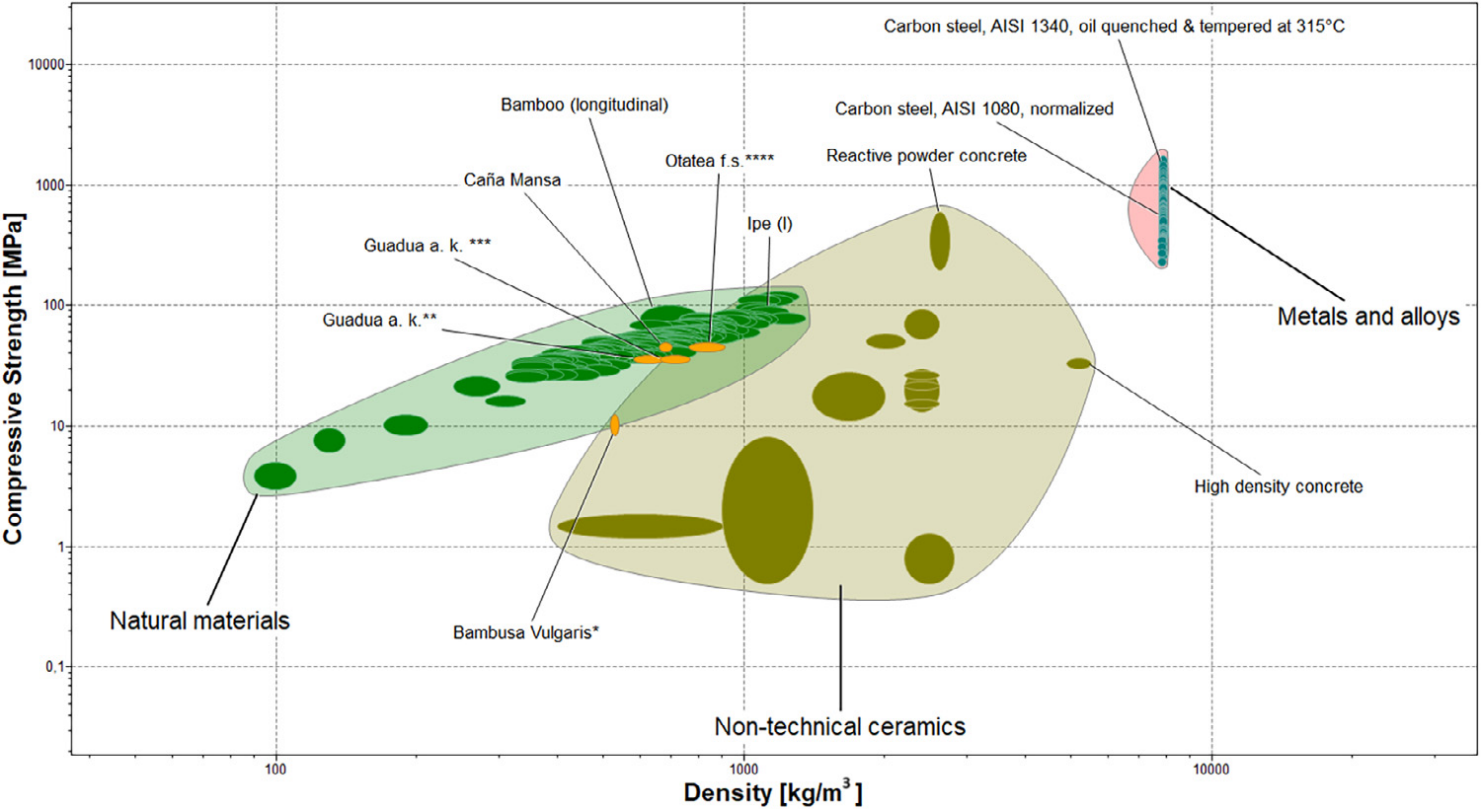
Material property chart created using CES EduPack 2019 for comparison of the compressive strength as a function of density among several structural materials. The minimum and maximum average compressive strength of the three culm sections of the Ecuadorian “Caña Mansa” bamboo biotype were added into the plot. Data styled from reference * [6], ** [7], *** [8], and **** [10].

**Fig. 9:**
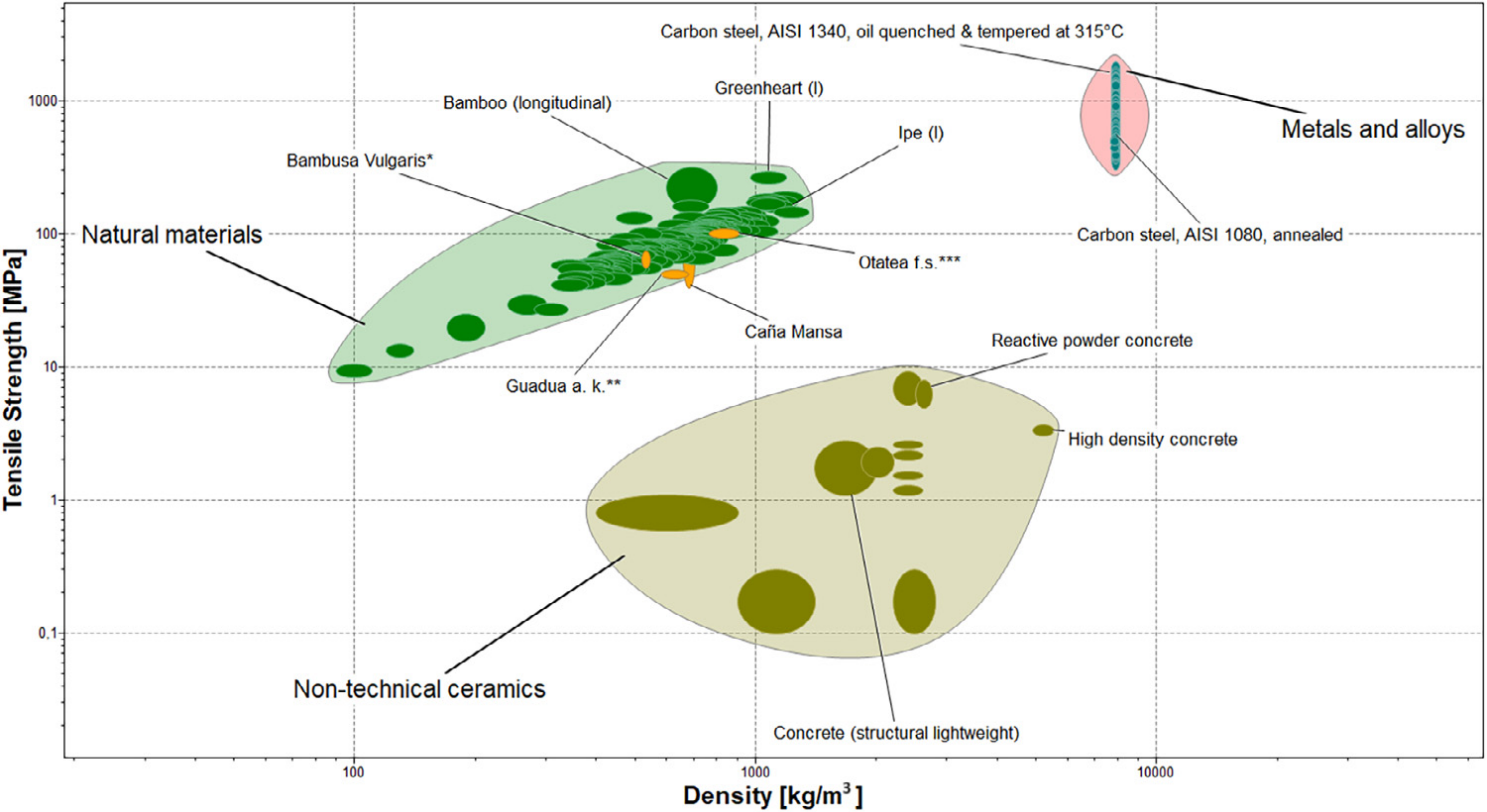
Material property chart built using CES EduPack 2019 for comparison of the tensile strength vs. density among structural materials. The minimum and maximum average tensile strength of the three culm sections of the Ecuadorian “Caña Mansa” bamboo biotype were also plotted. Data styled from reference * [6], ** [7], and *** [10].

**Fig. 10:**
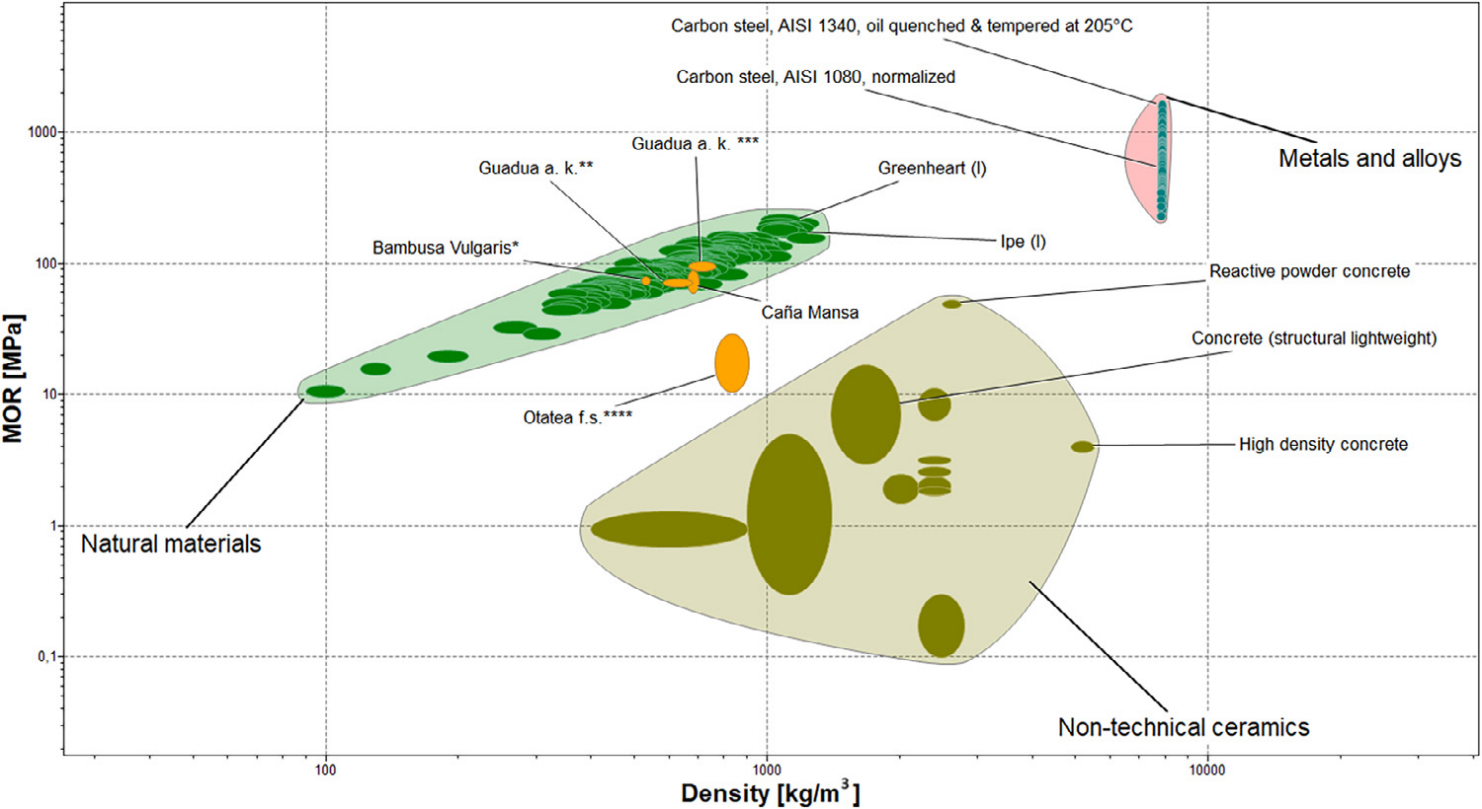
Material property chart built using CES EduPack 2019 for comparison of the MOR vs. density for structural materials. The minimum and maximum average MOR of the three culm sections of the Ecuadorian “Caña Mansa” bamboo biotype were incorporated into the plot. Data styled from reference * [6], ** [7], *** [8], and **** [10].

Finally, Fig. 8, Fig. 9, Fig. 10, show material property charts plotted using CES EduPack 2019 with the aim of comparing the compressive, tensile, and flexural strengths vs. density, respectively. Structural materials such as concrete, structural steel, and different bamboo species used in building applications were included in the charts, along with the “Caña Mansa” bamboo biotype. For each property, the minimum and maximum average strength of the three culm sections presented in Table 2, were used to plot the charts.

## 4 Experimental Design, Materials and Methods

The methods used for the characterization of the mechanical and physical properties of the Ecuadorian “Caña Mansa” bamboo culms were based on the ISO 22157:2019 standard, Parts 1 and 2 [1,2].

### 4.1 Material selection

The “Caña Mansa” was harvested from the province of Manabí, Ecuador, as shown in Fig. 1. Culms of approximately three years of age were selected from two clumps, called A and B. They were divided into three usable sections along their height, as illustrated in Fig. 2, and marked with a specific nomenclature described in Fig. 12. The material was then dispatched for further characterization.

### 4.2 Culms drying

The harvested culms were dried at atmospheric conditions in Quito before characterization. Their diaphragms were perforated to extract the internal water and reduce the drying time, which lasted about five months. During this time, the water content was monitored by using a COBOS moisture meter, model MD-4G, up to the moisture content achieved 20 % in three points along each culm section. Afterwards, the specimens were extracted from the culm and manufactured according to ISO 22157:2019 standard.

### 4.3 Manufacturing of accessories

**Fig. 11:**
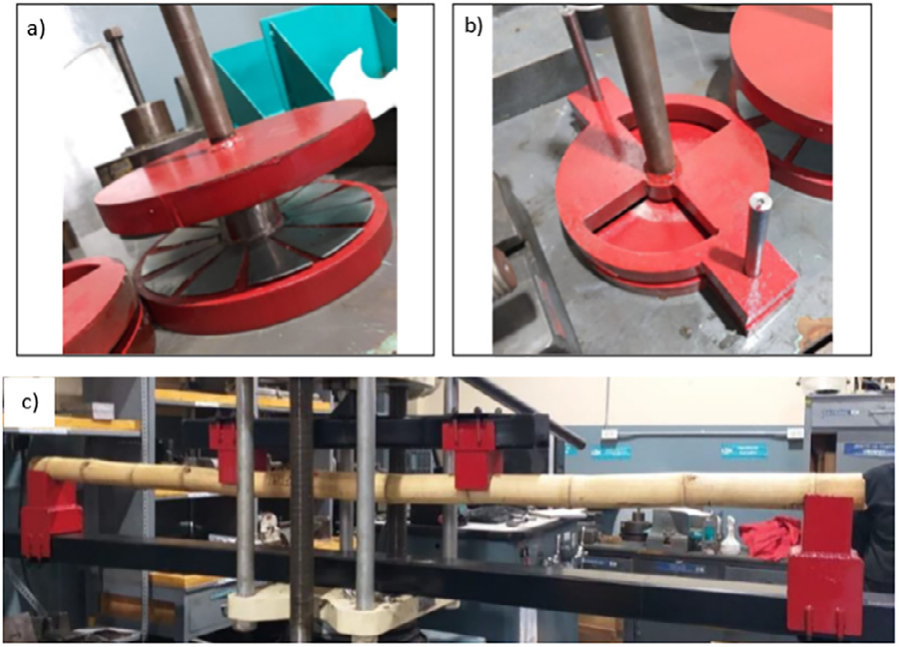
Accessories manufactured for performing a) compression, b) shear, and c) bending tests.

Accessories were manufactured to be coupled to the testing machine for the compression, shear and bending tests. These accessories were designed to satisfy the conditions detailed in the ISO 22157:2019 standard [1,2]. In the case of compression and shear tests, A36 steel plates were used. Steel tube rectangular beams were used for the main structure and supports in the case of the bending test. The manufactured accessories are displayed in Fig. 11. For the tensile tests, the standard accessories of the testing machine were used.

### 4.4 Specimen fabrication

The height of the specimen for compression and shear tests must be equal to its average outer diameter. Since a bamboo culm is not a perfect cylinder, and its diameter decreases as it moves away from the base, the average diameter was obtained by measuring four diameters from the region previously selected for fabrication. According to ISO 22157:2019 standard [1,2], the compression test on specimens with nodes is optional; therefore, only specimens of the internode region (see Fig. 2) were manufactured. On the other hand, the same number of specimens for shear and tensile tests were fabricated from the node and internode region.

The specimens for tensile tests have a rectangular flat section (see Fig. 3c), where the thickness varies depending on the wall of the culm section and the material removed to eliminate the curved surface to prevent failures and slippage in the gripping area. It is worth mentioning that the material was removed in the same proportion towards the middle section of the wall, meaning that the tensile strength belongs to this zone.

The bending test specimen is a piece of the culm, with the length equal to 30 times the diameter. Four diameters were measured in each end to obtain the average outer diameter. It is important to ensure that the specimens exhibit symmetrical spacing between nodes to properly distribute the applied load during the test. The specimens’ geometry is detailed in Fig. 3d.

Finally, the samples used for the physical tests were taken from the fractured specimens, after the mechanical tests (see Fig. 4).

### 4.5 Identification of specimens

**Fig. 12:**
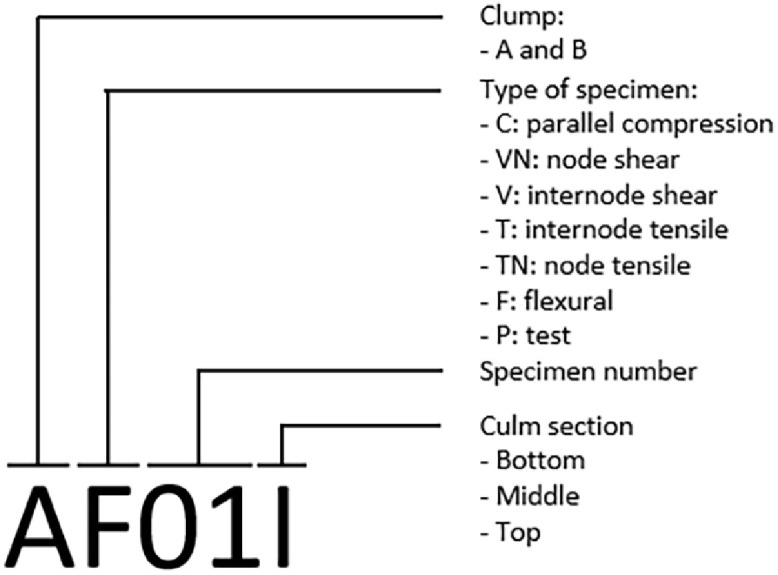
Nomenclature of specimens detailed in the repository file.

The identification was carried out with a specific nomenclature indicating the clump (A and B) and height section of the culm (bottom, middle or top) from which they were extracted, the number and type of specimen. The nomenclature of specimens is presented in Fig. 12 and was used in data described in the repository file.

### 4.6 Mechanical tests

The compression, shear and bending tests were carried out at the Laboratory of Stress and Vibration Analyses (LAEV) of the Faculty of Mechanical Engineering at EPN. Tensile, moisture content and density tests were performed at the Laboratory of Materials, Soils and Rock Testing (LEMSUR) of the Faculty of Civil and Environmental Engineering at EPN. At LAEV, a Tinius Olsen Super L-120 universal testing machine was used. A Shimadzu UH-F500kNX universal testing machine was employed at LEMSUR.

The shear and compression tests require a maximum preload of 1 kN to settle the specimen. Subsequently, the load is applied continuously to maintain a constant head speed of 0.6 mm/min, and the maximum load at which the specimen fails is recorded. The test is not halted until the failure is most pronounced.

In the tensile test, the gauge portion is measured with a resolution of 0.1 mm at three different points, and an average value is calculated. Additionally, finely knurled grips were used to prevent slippage in the gripping area. The load is applied continuously and at a constant head speed of 0.6 mm/min. The test is not stopped until the specimen separates into two parts.

In the bending test, the specimen was placed on the supports of the lower fixture, allowing it to assume a natural position. Next, the upper fixture was positioned, and all components in this test were aligned in a vertical plane to ensure that the load is distributed correctly and continuously applied at a constant speed of 30 mm/min. The maximum load at which the specimen failed was recorded, but the test was not finished until the failure was most pronounced.

### 4.7 Physical tests

The density and moisture content tests were conducted at LEMSUR. To determine the moisture content, the samples were extracted from each tested specimen, preferably from the section nearest to the failure that occurred during mechanical testing. The sample dimensions were 25 × 25 mm, with a thickness equal to that of the wall of the test specimen. First, the mass was measured using a digital balance UX 1020H model with an accuracy of 0.001 g, to obtain m (mass of the test sample before drying). Subsequently, the samples were placed in a BIOBASE BOV-D30 electric oven at a temperature of 103±2°C for 24 h. Once this time was completed, the mass was recorded. If the difference between successive mass determinations does not exceed 0.01 g, the sample is considered in a dry state, $m_{o}$ (mass of the test sample after drying) is recorded, and the moisture content can be determined using Eq. (5). Otherwise, the samples need to be reintroduced in the oven at two-hour intervals until the mentioned mass difference is achieved to reach the dry state. The experimental raw data to determine the moisture content of each sample are detailed in the repository file.

According to ISO 22157 standard, samples for determining basic density are extracted from the zone nearest to the failure and must be dried in an oven to minimize shrinkage. Therefore, the same samples used in the moisture content test were employed for density measurements after they reached the dry state to record $m_{o}$, the mass of the test sample after drying. The volume of the dried samples was obtained by measuring the sample dimensions with a vernier caliper, considering a rectangular prism shape. Finally, the density was computed by Eq. (6). It is worth noting that the surface curvature for the sample configuration (shape and dimensions) was minimal and practically did not influence the volume calculation. As a matter of fact, preliminary tests were performed using Archimedes’ method to verify the density of samples, and no difference was found with the geometrical method.

In the moisture content and basic density tests, 44 samples (extracted from the tested specimens as aforesaid) were replicated for each culm section (bottom, middle, and top). In other words, the analysis of the results was conducted considering each section of the culm to ascertain whether there is a significant variation in moisture content and density at different heights.

A set of articles was used to collect density and strength data of other structural bamboo species, which were compared with the “Caña Mansa” biotype taking into account the three sections of the culm and the standard deviation of the results, as shown in Figs. 5 and 6.

## Limitations

None.

## CRediT authorship contribution statement

**José Padilla:** Conceptualization, Investigation, Validation, Writing – original draft. **Wilson Guachamín-Acero:** Conceptualization, Supervision, Data curation. **Víctor H. Guerrero:** Formal analysis, Writing – review & editing. **Willan Monar:** Conceptualization, Supervision, Formal analysis. **Patricia I. Pontón:** Methodology, Data curation, Writing – review & editing. **Marco V. Guamán:** Conceptualization, Supervision, Formal analysis, Writing – review & editing.

## Data Availability

Dataset on the mechanical and physical characterization of the Ecuadorian Guadua angustifolia kunth bamboo culms belonging to “Caña Mansa” biotype (Original data) (Mendeley Data).
